# Contraction and Expansion of Nanocomposites during Ion Exchange Reactions

**DOI:** 10.1021/acs.cgd.1c01364

**Published:** 2022-03-14

**Authors:** Arno van der Weijden, Martin van Hecke, Willem L. Noorduin

**Affiliations:** † AMOLF, Science Park 104, Amsterdam 1098 XG, The Netherlands; ‡ Leiden Institute of Physics, Leiden University, Niels Bohrweg 2, Leiden 2333 CA, The Netherlands; § Van ‘t Hoff Institute for Molecular Sciences, University of Amsterdam, Science Park 904, Amsterdam 1090 GD, The Netherlands

## Abstract

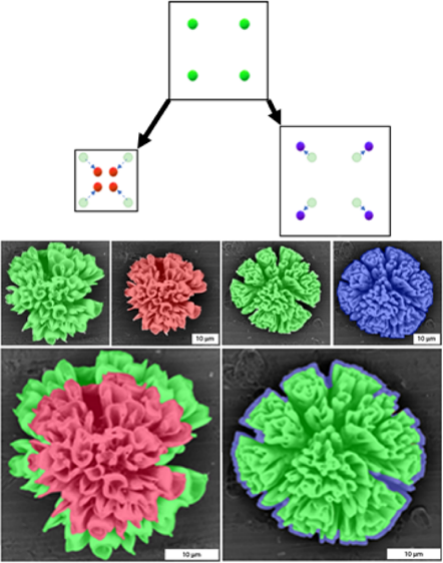

The next generation of advanced functional materials can greatly benefit from methods for realizing the right chemical composition at the right place. Nanocomposites of amorphous silica and metal carbonate nanocrystals (BaCO_3_/SiO_2_) form an attractive starting point as they can straightforwardly be assembled in different controllable three-dimensional (3D) shapes, while the chemical composition of the nanocrystals can be completely converted via ion exchange. Nevertheless, it is still unknown—let alone predictable—how nanoscopic changes in the lattice volume of the nanocrystals translate to changes in the microscopic dimensions of 3D BaCO_3_/SiO_2_ structures during ion exchange. Here, we demonstrate that the microscopic shape adapts to contraction and expansion of the atomic spacing of nanocrystals. Starting from BaCO_3_/SiO_2_, we systematically decrease and increase lattice volumes by converting the BaCO_3_ nanocrystals into a range of chalcogenides and perovskites. Based on geometrical analysis, we obtain a precise prediction for how the microscopic nanocomposite volume follows the change in nanoscopic crystal volume. The silica matrix facilitates mechanical flexibility to adapt to nanoscopic volume changes, while preserving the 3D morphology and fine details of the original composite with high fidelity. The versatility and predictability of shape-preserving conversion reactions open up exciting opportunities for using nanocomposites as functional components.

## Abstract 2

Shape-controlled nanocomposites of barium carbonate nanocrystals and amorphous silica (BaCO_3_/SiO_2_) are converted into a range of chalcogenides and perovskites to systematically decrease or increase the lattice volume. Geometrical analysis shows that the microscopic nanocomposite volume closely follows changes in the nanoscopic crystal volume, opening up exciting opportunities for using nanocomposites as functional components.

## Introduction

Self-assembly offers exciting opportunities for the bottom-up ordering of advanced functional nano- and micromaterials.^1−11^ Nanocomposites composed of barium carbonate nanocrystals that are embedded in an amorphous silica matrix (BaCO_3_/SiO_2_) are an ideal platform to exploit this potential. These composites, also known as biomorphs, straightforwardly form in an acid-regulated coprecipitation.^12−25^ A wide diversity of three-dimensional shapes such as corals, vases, and helices can straightforwardly be formed. In addition, more refined sculpting and patterning are possible by controlling the reaction conditions such as pH, temperature, and carbonate concentration during the coprecipitation to pattern, sculpt, and hierarchically organize these composites.^16,17^

The coprecipitation mechanism inherently restricts the chemical compositions that are suitable for this self-assembly process to barium, strontium, and calcium carbonate salts that precipitate together with silica. Since carbonate salts and silica have limited application potential, post-assembly methods have been developed to modify the chemical composition of the nanocomposites. For example, the silica matrix has been modified with chemical groups and nanoparticles.^19,20^ Moreover, the chemical composition of the carbonate nanocrystals can be completely altered by sequentially exchanging both cations and anions while preserving the shape of the original composite.^26−34^ Already, such shape-preserving exchange reactions have been developed toward a wide selection of chemical compositions including metal halide perovskites, metal chalcogenides, and metals.^26,27,29,30,35^ Moreover, the first functional materials that show mechanical actuation and tunable catalytic activity have been developed by exploiting the independent control over the shape, structure, and composition.^27,29^

**Figure 1:**
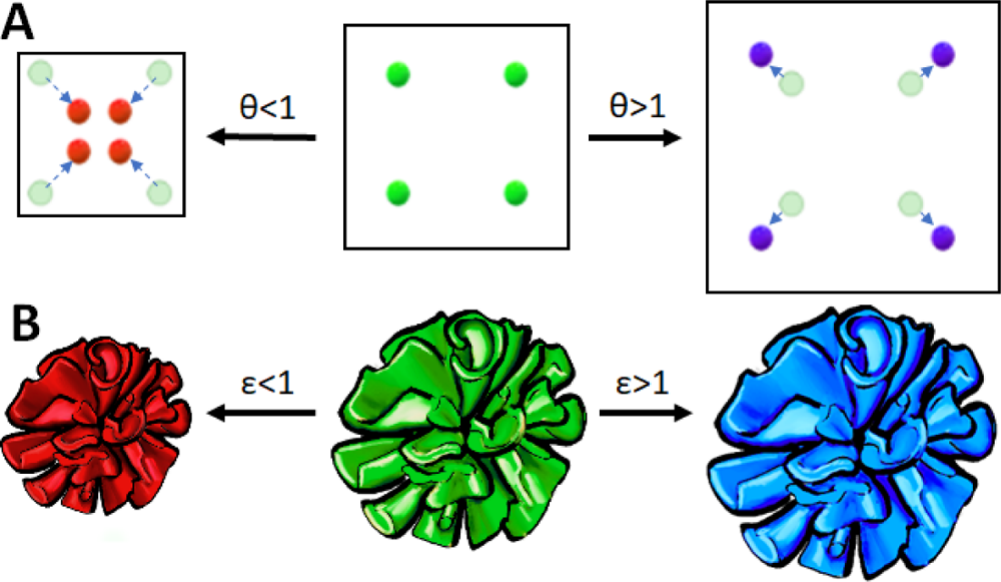
(A) Schematic drawing of contraction and expansion of nanocomposites during ion exchange reactions. During ion exchange, the crystal lattice volume ratio (θ) decreases (θ < 1) or increases (θ > 1). (B) Schematic drawing showing that decreasing and increasing crystal lattice volumes translate to contracting (ε < 1, red) and expanding (ε > 1, blue) microscopic volumes of the nanocomposite, respectively.

While the conversion reactions on the nanocomposites are material-agnostic and the overall shape is preserved, a change in microscopic size has been observed.^27^ Importantly, as anions and cations with very different atomic radii are exchanged, the nanocrystals undergo changes in the lattice spacings, which we quantify by the crystal lattice volume ratio θ, which is unequal to 1 (Figure 1A).^27^ These nanoscopic changes influence the microscopic dimensions of the 3D shape (Figure 1B). However, it is not known how the crystal lattice volume ratio θ impacts the volume ratio of the nanocomposite (ε), if the composite accommodates both expansion and contraction of the crystal lattice, and to what extent the reaction conditions play a role. Here, we systematically investigate and obtain a precise prediction for the final volume change in these heterogenous structures.

## Results and Discussion

**Figure 2:**
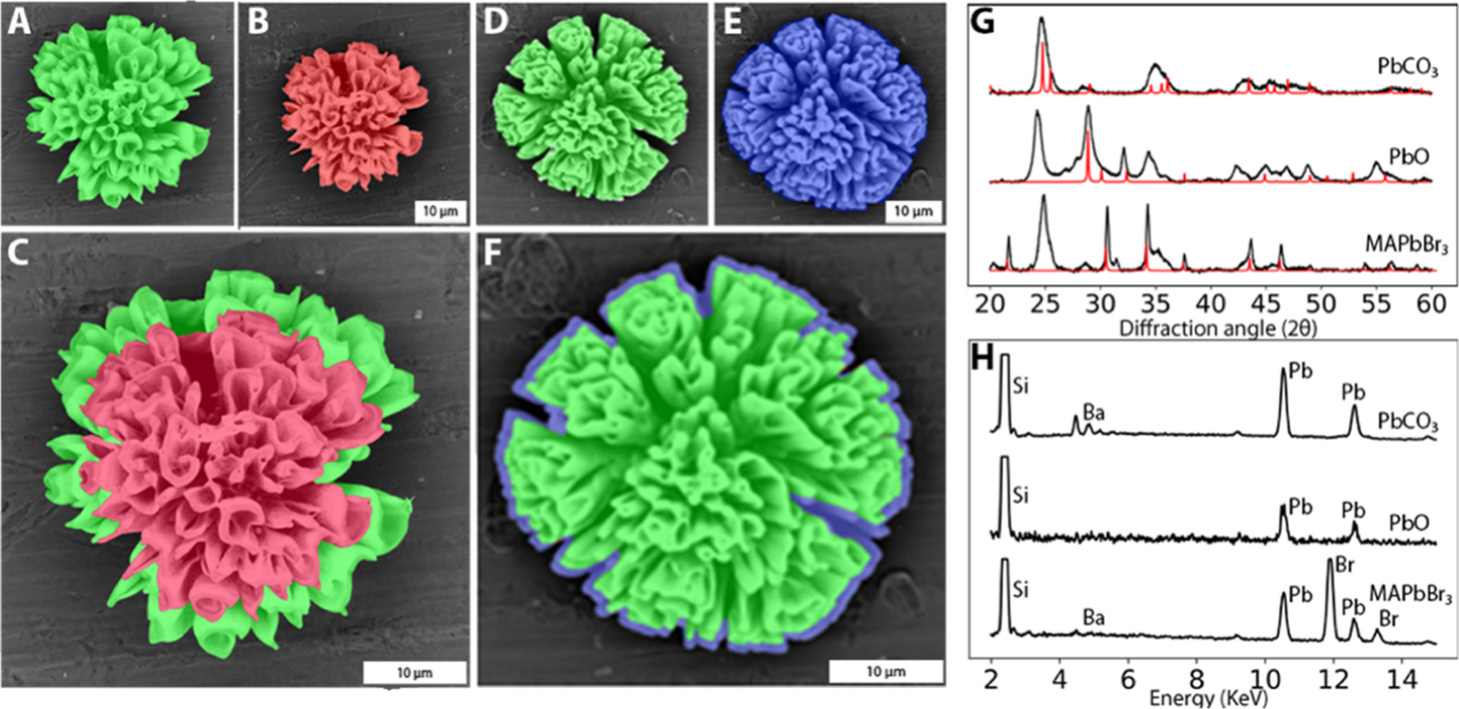
Orthographic SEM images of the same composites before and after conversion to a smaller or bigger nanocrystal (false color indicates the original structure (green), reduction (red), and increase (blue) in the crystal lattice volume). (A) SEM before and (B) after conversion from BaCO_3_ (green) to PbO (red). (C) Overlay image shows a decrease in the microscopic shape. (D) SEM before and (E) after conversion from BaCO_3_ (green) to MAPbBr_3_ (blue). (F) Overlay image shows an increase in the microscopic shape. (G) XRD analysis shows the crystallographic conversion, reference peaks indicated in red.^36^ (H) EDS analysis shows full chemical conversion.

To illustrate the nature of the problem, we perform conversion reactions for two cases corresponding to shrinking and expansion. We define the adjusted unit cell ratio as
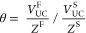
1with *V*_UC_^S^ and *V*_UC_^F^ as the unit cell volumes of the starting material and final material, respectively, and *Z*^S^ and *Z*^F^ as their respective number of formula units per cell. Specifically, using previously developed methods,^17^ we first precipitate coral-like BaCO_3_/SiO_2_ nanocomposites and convert the BaCO_3_ nanocrystals into lead carbonate (PbCO_3_). If we thermally decompose the PbCO_3_ to lead oxide (PbO) at 380 °C under N_2_ (see the Supporting Information for details), we obtain θ = 0.52 (<1).^26^ Alternatively, if we expose PbCO_3_ to methyl ammonium bromide at 120 °C to produce methyl ammonium bromide perovskite (MAPbBr_3_), we obtain θ = 1.37 (>1). X-ray diffraction (XRD) confirms the crystallographic conversion,^36^ while energy dispersion spectroscopy (EDS) confirms complete chemical conversion to PbO and MAPbBr_3_ (Figure 2G,H).^26,27^

To investigate how θ impacts the volume ratio of the nanocomposite (ε), we characterize the microscopic morphology changes by comparing scanning electron microscopy (SEM) images before and after conversion of the same coral-like form (Figure 2A–F). We find that the original form is preserved with only minor deformations and that the nanocomposite shrinks and expands as expected. However, the relative volume ratio of a nanocomposite, ε, appears to be less different from 1 than expected from the changes in unit cell volume θ. Hence, the question is whether there is a unique relation between the two and if so, what this relation is.

**Figure 3:**
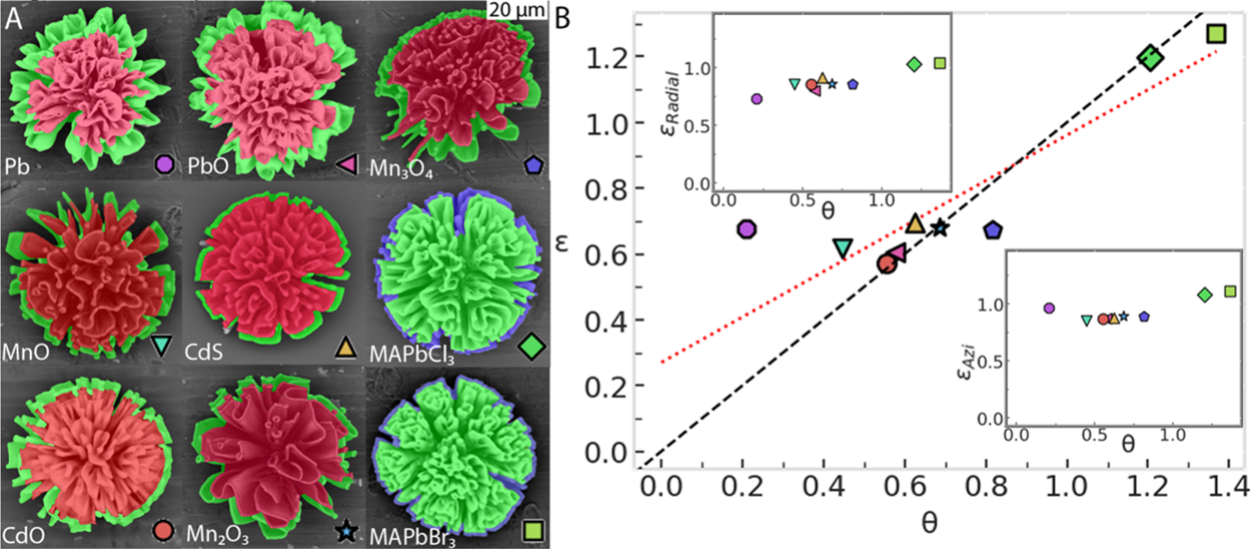
Systematic shrinking and expansion of the crystal lattice volume. (A) SEM images of the nanocomposites before (green) and after (red) conversion. (B) Total volume change of the structures ε, calculated as ε = ε_Radial_*ε_Azi_^2^, against the unit cell volume change θ.^27^ The insets show ε_Radial_ and ε_Azi_. The dashed black lines represent a slope of 1 between ε and θ. The dotted red line is a linear fit of the experimentally determined data with formula ε = 0.69*θ + 0.27, indicating that there is a direct relation between θ and the relative volume changes of a nanocomposite.

To answer this question, we perform conversion reactions toward a wide range of metal oxides, sulfides, and perovskites with a wide diversity of crystal volumes and determine microscopic morphology changes. Specifically, based on previously developed reaction conditions,^26−28^ we decrease θ by performing conversion reactions toward metallic lead (Pb, θ = 0.21), manganese monoxide (MnO, θ = 0.45), cadmium oxide (CdO, θ = 0.56), lead monoxide (PbO, θ = 0.58), cadmium sulfide (CdS, θ = 0.62), dimanganese trioxide (Mn_2_O_3_, θ = 0.69), and trimanganese tetraoxide (Mn_3_O_4_, θ = 0.82) (Figure 3A). Moreover, we increase the crystal lattice volume by performing conversion reactions toward methylammonium lead chloride (MAPbCl_3_, θ = 1.20) and methylammonium lead bromide (MAPbBr_3_, θ = 1.37), to ascertain the adaptability of the nanocomposite layout for a wide variety of crystallographic volume changes (Figure 3A).

To compute ε, we need to access the volumes of the nanocomposites. As we only have access to 2D SEM images, we proceed as follows. First, for all reactions, we determine the radial (ε_Radial_) and azimuthal (ε_Azi_) length ratios from the SEM images. SEM analysis shows that coral-like forms remain approximately half-spherical after conversion (see Supporting Information Figure S16). Moreover, the composites are only attached to the substrate at the nucleation point in the center, and the nanocrystals are all aligned radially away from this center. Therefore, we can assume a hemispherical symmetry for the coral-like shapes (see Supporting Information Section 19).^27^ Based on this hemispherical symmetry, ε_Azi_ is determined from the distance between the left and right points of extremities in the coral shape, while ε_Radial_ is calculated from the distance of each extremity to the center of the half-sphere (Figure 3B, insets. See Supporting Information Section 18).^27^ We compute the volume ratio ε as
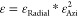
2

A scatter plot of the unit cell ratio θ versus ε shows good data collapse, meaning that there is a direct relation between θ and the relative volume changes of a nanocomposite (Figure 3B). While this relation is approximately linear, so that shrinking and expansion of the nanocrystals directly impact the volume of the composite, the slope of the linear fit is less than 1 (ε = 0.69*θ + 0.27), consistent with our observations in Figure 2.

To understand the difference between θ and ε, we consider the role of the silica matrix. The crucial insight is that this matrix maintains a constant distance from the nanocrystals and does not change volume during conversion—only the nanocrystals do. Hence, based on this, we should not expect θ and ε to be equal. To incorporate the role of the silica matrix, we proceed as follows. First, we decompose the volume of the nanocomposite before conversion as *V*_Comp_^S^ = *V*_NC_^S^ + *V*_SiO2_^S^, where *V*_NC_^S^ and *V*_SiO2_^S^ are the volumes of the nanocrystals and matrix at the start of the conversion, respectively. Second, we assume *V*_SiO2_^S^ = λ*V*_Comp_^S^, where λ is the volume fraction of the matrix before conversion, which is calculated from the atomic ratios and densities of the BaCO_3_ nanocrystals and silica to be 0.20 ± 0.03 (see Supporting Information Section 17 for details).^14,27^ We then assume that the silica matrix adapts its shape to follow the volume changes of the nanocrystal inclusions, so no significant gaps between the crystal and matrix are introduced during conversion. Third, we also decompose the volume of the nanocomposite after conversion as *V*_Comp_^F^ = *V*_NC_^F^ + *V*_SiO2_^F^, where *V*_NC_^F^ and *V*_SiO2_^F^ are the volumes of the matrix and nanocrystals when conversion is finalized. Since the matrix volume should not change during conversion, we assume *V*_SiO2_^S^ = *V*_SiO2_^F^. Combining these equations yields the following two expressions: *V*_Comp_^F^ = *V*_NC_^F^ + *V*_SiO2_^S^ and . Based on this, we derive the silica-adjusted volume ratio (φ) as
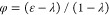
3

**Figure 4:**
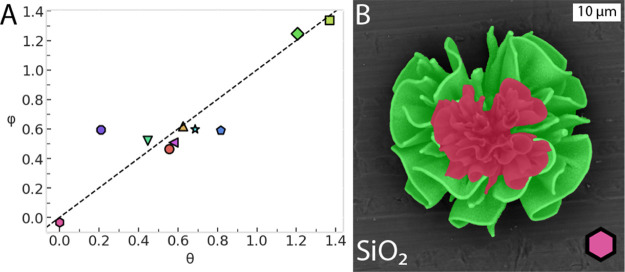
Influence of the silica matrix on shape adaptability during ion exchange. (A) Silica-adjusted volume ratio φ (=(ε – λ)/(1 – λ)) plotted against the formula unit adjusted unit cell volume ratio θ. Note that compared to Figure 3B, this plot includes an extra datapoint at θ = 0 for a conversion in which all nanocrystals are removed. (B) Complete removal of nanocrystals from CdCO_3_/SiO_2_ (green) results in a silica structure (red, hexagon symbol in Figure 4A), which albeit shrunken more than five times still exhibits the form and fine details of the original composite, demonstrating shape preservation and the adaptability of the silica matrix.

We can also rewrite eq 3 as ε = (1 – λ)θ + λ, which for λ = 0.2 is in good agreement with the slope found in Figure 3B (ε = 0.69θ + 0.27). As a final validation, plotting φ against θ collapses into a linear plot, hence showing that φ is indeed approximately equal to θ (Figure 4A). This indicates that our assumption that the silica matrix adapts is warranted.

To further validate our description, we probe the influence of the reaction conditions on the adaptability of the silica matrix. It is known that the presence of H_2_ and the temperature can affect the flexibility of silica.^37^ As we conduct reactions with MnO and Pb in the presence and absence of H_2_ over a wide range of temperatures (120–500 °C), we indirectly probe the adaptability of the matrix. We notice that for the specific case of Pb, the shrinking is less than expected, which is consistent with a matrix that would be less flexible due to the presence of H_2_ during conversion. For all other conversions, no significant deviations are found, and the matrix adapts nearly perfectly. For the CdO reaction, we systematically vary the temperature between 250 and 500 °C and did not observe any significant change in volume (see the Supporting Information). Hence, despite the wide range of reaction conditions, the assumption of a perfectly adapting matrix holds well, which implies that our description is to a large extent reaction condition-agnostic.

Finally, we investigate the adaptability of the matrix in the extreme case that the nanocrystals are completely removed. If our prediction is correct, this should lead to an extreme shrinkage ratio of the structure equaling to ε = λ, as λ is the volume fraction of *V*_SiO2_^S^ in *V*_Comp_^S^. To this aim, we remove the nanocrystals from the composite by first converting the nanocrystals to CdO and subsequently removing them via sublimation at 250 °C to minimize mechanical deformation of the silica matrix caused by surface tension effects. EDS analysis confirms that all CdO is removed and only a silica matrix is retained. SEM analysis before and after conversion shows a drastic shrinkage of the structure’s size where the volume ratio approximates ε = 0.17, which is approximately equal to λ = 0.2, and that the silica-adjusted volume ratio approximates zero (φ = −0.03) (Figure 4B). Surprisingly, even after this more than fivefold reduction in volume, the overall shape is mostly preserved. Especially, the sub-micrometer fine surface features remain almost unchanged. This showcases the adaptability and flexibility of this SiO_2_ matrix.

## Conclusions

Nanocrystals in shape-controlled composite materials can straightforwardly be converted into many different chemical compositions by sequential ion exchange reactions. The exchange of ions results in volume changes of the crystal lattice (θ). Here, we show how θ translates to volume changes in microscopic nanocomposites (ε). To this aim, we define the silica-adjusted volume ratio (φ) to account for the inert silica matrix and perform a geometrical analysis on the composites before and after conversion. It should be noted that this analysis does not take into account internal nanoscopic processes such as incorporation of silica, thermal migration during annealing, and changes in the interfaces and nanoparticle size changes that may influence θ.^38^ However, even while deliberately simplifying the internal processes using literature values of θ, we already find that the microscopic volume predictably adapts to the nanoscopic volume while preserving the fine features and overall 3D shape of the original composite.

Our results highlight that the silica matrix in the composite facilitates shape-preserving conversions, not only by enabling diffusion of ions in and out of the composite for rapid ion exchange^27^ but also by providing both mechanical stability and adaptability to accommodate the volume changes of the nanocrystals. This paradoxical combination of mechanical stability and chemical reactivity is thus essential for conversion of nanocomposites into a large diversity of chemical compositions. The flexibility of the silica matrix renders an adaptable scaffold to facilitate even dramatic swelling and shrinking of the microscopic form while preventing cracking or mechanical failure to enable excellent shape preservation and predictable volume changes. By removing the nanocrystals, we achieve fivefold shape-preserving volume shrinkage, suggesting a route toward extreme miniaturization. The scaffold has such an adaptability that if a reaction on the nanocrystals is possible, the nanocomposite will likely accommodate the volume changes for shape preservation.

We foresee that these insights may directly impact our ability to shape chemical compositions according to an exact design by first programming a desired shape and subsequently customizing the chemical composition. As a first step, we recently demonstrated that photogeneration of carbonate enables light-controlled nucleation and sculpting of these composites.^39^ The next step toward this ambitious goal will be to gain complete hands-on control over the self-assembly process of the nanocomposite in three dimensions, thus opening new routes toward rationally designed functional structures for catalysis, optics, and photovoltaics.

## Supporting Information Available

Synthesis methods and used equipment (PDF)

## Abbreviations

XRD: X-ray diffraction
EDS: energy-dispersive X-ray spectroscopy
SEM: scanning electron microscopy
IR: infrared spectroscopy
