# Supplementary material for: Contraction and Expansion of Nanocomposites during Ion Exchange Reactions
Source: Cryst Growth Des. 2022 Mar 14;22(4):2289–93. doi: 10.1021/acs.cgd.1c01364 (PMC8990519; doi:10.1021/acs.cgd.1c01364)
Supplement: Supplementary file 1 — cg1c01364_si_001.pdf [file cg1c01364_si_001.pdf]

**Supporting Information**

**Contraction and expansion of nanocomposites during ion exchange reactions**

Arno van der Weijden[1], Martin van Hecke[1,2*], Willem L. Noorduin[1,3*]
[1] *AMOLF, Science Park 104, 1098XG Amsterdam, The Netherlands*
[2] *Leiden Institute of Physics, Leiden University, Niels Bohrweg 2, Leiden 2333 CA, The Netherlands*
[3] *Van 't Hoff Institute for Molecular Sciences, University of Amsterdam, Science Park 904, Amsterdam 1090 GD, The Netherlands.*

**S1. Growth of $BaCO_3/SiO_2$ nanocomposites**

$BaCO_3/SiO_2$ nanocomposites on a substrate were prepared as described in previous literature.[1] In short, a substrate (e.g., 2x2 cm slide of aluminium) was vertically positioned in a 100 mL beaker containing $BaCl_2$ dihydrate (74 mg, 0.3 mM) and $Na_2SiO_3$ (16 mg, 0.13 mM) dissolved in 15 mL of water. The reaction vessel was loosely covered with a Petri dish to allow $CO_2$ from the air to slowly diffuse into the reaction mixture. Typical growth times ranged between 1.5-2.0 hours after which the substrate was removed from the solution and washed with deionized water. The resulting $BaCO_3/SiO_2$ nanocomposites are then instantly converted in the next step.

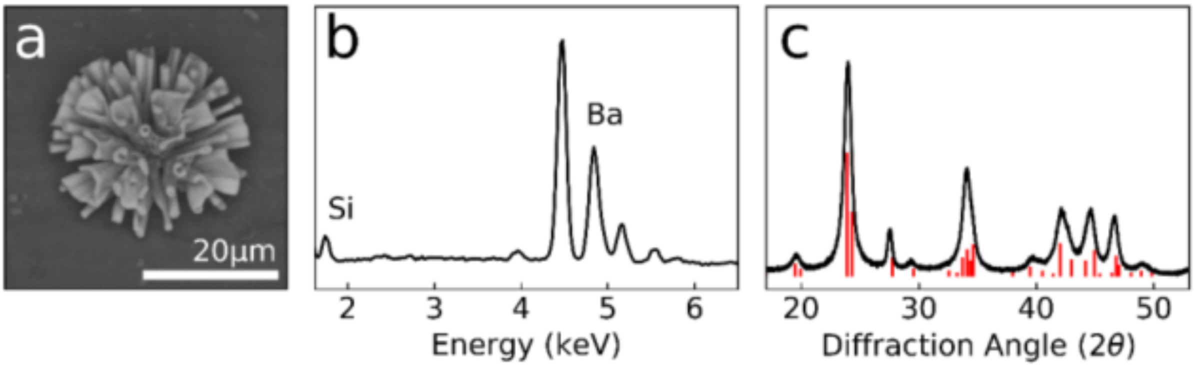


Figure S1: $BaCO_3$ nanocomposites. (A) SEM, (B) EDS, (C) XRD with reference lines depicted (COD 1000033). [1]

**S2. Scaled up growth of $BaCO_3/SiO_2$ nanocomposites**

A solution of $BaCl_2$ (7.4 g, 30 mM) in 300 mL of water was added to a solution of $Na_2SiO_3$ (1.6 g, 13 mM) in 1200 mL of water. This solution was shortly stirred and poured in a metal tray (30x50x10 cm) to maximize the surface area in contact with air, while keeping at least 1 cm of depth to the solution. The solution was left for 1.5 hours with the tray covered polycarbonate lid which is perforated with 0.3mm holes. The resulting nanocomposites floating on the meniscus were separated from the solution via vacuum filtration. For further exchange reactions, the microstructures were instantly removed from the filtration paper with a spatula and transferred directly into the exchange solution.

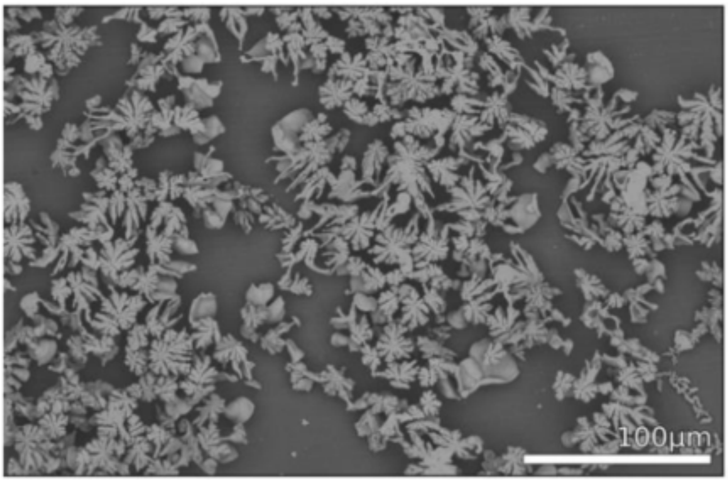


Figure S2: SEM of $BaCO_3/SiO_2$ nanocomposites grown on the meniscus.[1]

**S3. Conversion to $PbCO_3$ nanocomposites**

Lead nitrate ($Pb(NO_3)_2$) (15 g, 906 mM) was dissolved in 50 mL degassed and demineralized water under a nitrogen atmosphere. A substrate containing fresh $BaCO_3$ nanocomposites was placed in this solution for 30-60 seconds. Afterwards, the substrate containing the white nanocomposites was removed and washed in three demineralized water baths followed by an acetone bath. Full exchange while preserving shape was determined with SEM and EDS. XRD showed that $PbCO_3$ was formed (Figure 2) (COD 1010956).

**S4. Conversion to $MAPbBr_3$ nanocomposites**

$PbCO_3$ nanocomposites were placed in a single zone tube furnace with methyl ammonium bromide (MABr) in an alumina boat placed upstream. The pressure was reduced to less than 5 mbar. The system was then purged with nitrogen gas ($N_2$) until atmospheric pressure was reached. This was repeated 5 times to purge all oxygen from the reaction chamber. The furnace was subsequently heated up to 120°C while the pressure was kept at 65 mbar with a $N_2$ flow of 30 sccm. After 6 hours the furnace was allowed to cool and the resulting nanocomposites were analysed with SEM, EDS and XRD (Figure 2) (ICSD 252415).

**S5. Conversion to $MAPbCl_3$ nanocomposites**

$PbCO_3$ nanocomposites were placed in a single zone tube furnace with methyl ammonium chloride (MACl) in an alumina boat placed upstream. The pressure was reduced to less than 5 mbar. The system was then purged with nitrogen gas ($N_2$) until atmospheric pressure was reached. This was repeated 5 times to purge all oxygen from the reaction chamber. The furnace was subsequently heated up to 120°C while the pressure was kept at 65 mbar with a $N_2$ flow of 30 sccm. After 6 hours the furnace was allowed to cool and the resulting nanocomposites were analysed with SEM, EDS and XRD (Figure S3). Note that for this sample XRD has been performed on lose structures on an aluminum slide and that XRD confirms full conversion for these well separated structures.

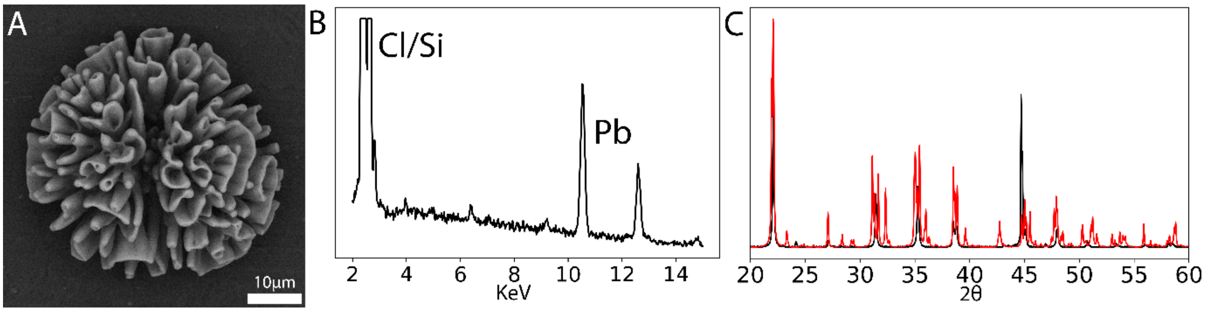


Figure S3: $MAPbCl_3$ nanocomposites. (A) SEM, (B) EDS, (C) XRD (10.1021/acs.jpcc.1c06835).

**S6. Conversion to PbO nanocomposites**

PbO nanocomposites were placed in a single zone tube furnace. The furnace was purged of oxygen and carbon dioxide and filled with nitrogen gas. The furnace was subsequently heated up to 380°C at atmospheric pressures with a ramp of 6°C/min. After 12 hours the furnace was allowed to cool and the resulting nanocomposites were analysed with SEM, EDS and XRD (Figure 2) (ICSD 40180).

**S7 Conversion to Pb nanocomposites**

PbO nanocomposites were placed in a single zone tube furnace. The furnace was purged of oxygen and filled with hydrogen gas till atmospheric pressures were reached. The furnace was subsequently heated up to 270°C to avoid melting the generated lead particles. After 48 hours the furnace was allowed to cool and the resulting nanocomposites were analysed with SEM, EDS and XRD (Figure S4). Note that conversions are for XRD are performed on large amounts of powder causing incomplete conversion. Individual structures do convert fully.

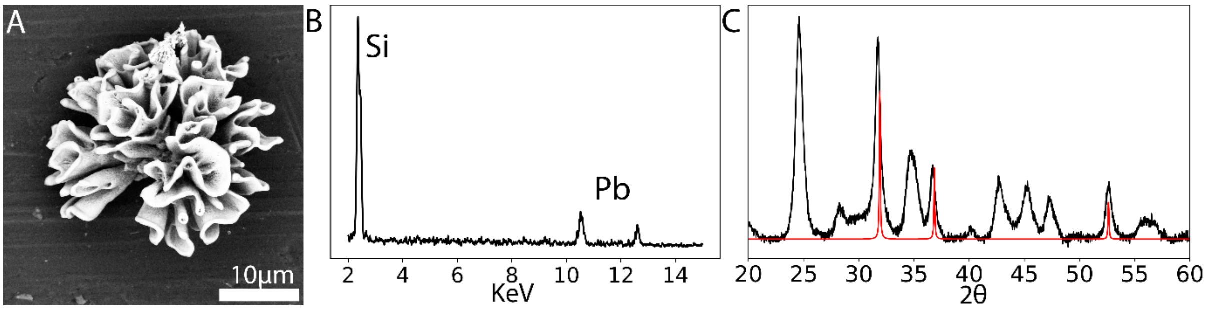


Figure S4: Pb nanocomposites. (A) SEM, (B) EDS, (C) XRD.

**S8 Conversion to $CdCO_3$ nanocomposites**

Cadmium chloride (458 mg, 50mM) was dissolved in 50 mL demineralized water. A substrate containing fresh $BaCO_3$ nanocomposites was placed in the solution for 12 minutes. The resulting $CdCO_3$ nanocomposites were washed in two demineralized water baths followed by an acetone bath. The structures were analysed using SEM, EDS and XRD (Figure S5).

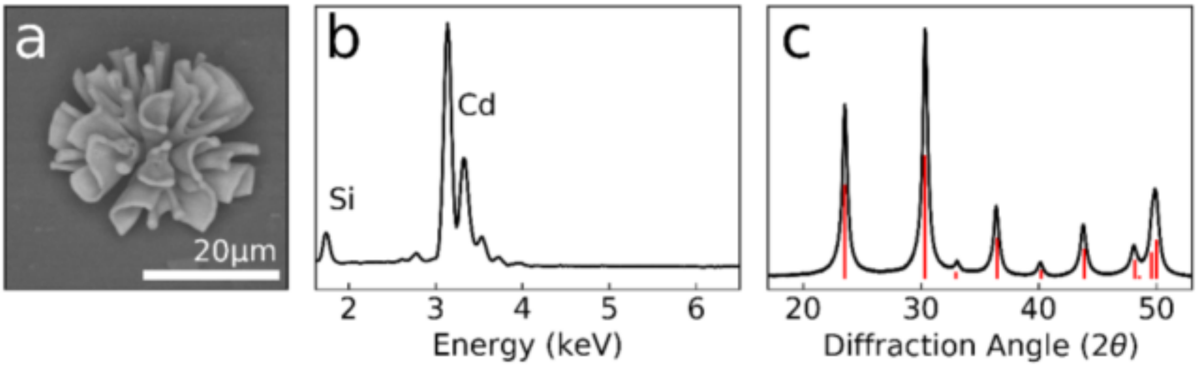


Figure S5: $CdCO_3$ nanocomposites. (A) SEM, (B) EDS, (C) XRD with reference lines depicted (AMCSD 0004324).[1]

**S9 Conversion to CdO nanocomposites**

$CdCO_3$ nanocomposites were placed in a single zone tube furnace. The furnace was purged of oxygen and filled with nitrogen gas till a pressure of 500 mbar was reached. This pressure was maintained with a 50 sccm flow of $N_2$ as the furnace was heated to 290°C for 4 hours. The resulting nanocomposites were characterized by SEM, EDS and XRD (Figure S6).

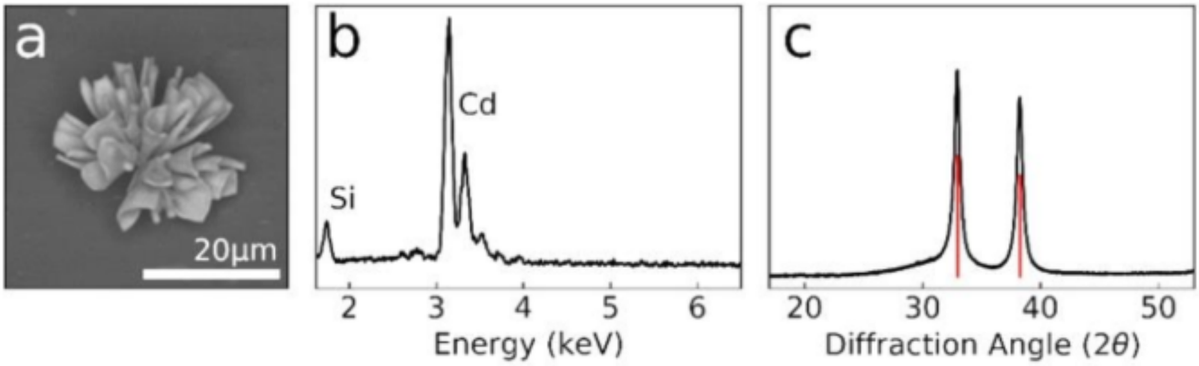


Figure S6: CdO nanocomposites. (A) SEM, (B) EDS, (C) XRD with reference lines depicted (COD 1011003).[1]

**S10 Conversion to CdS nanocomposites**

An aluminium substrate containing $CdCO_3$ nanocomposites was placed in a single zone tube furnace. The furnace was purged of oxygen and filled with argon and subsequently heated to 290°C. Upon reaching a stable temperature $H_2S$ (10 ml min$^{-1}$) and argon (90 ml min$^{-1}$) were flowed into the tube furnace for 45 minutes. Afterwards the furnace was allowed to cool while argon was flushed through it. The resulting CdS nanocomposites were characterized by SEM, EDS and XRD (Figure S7).

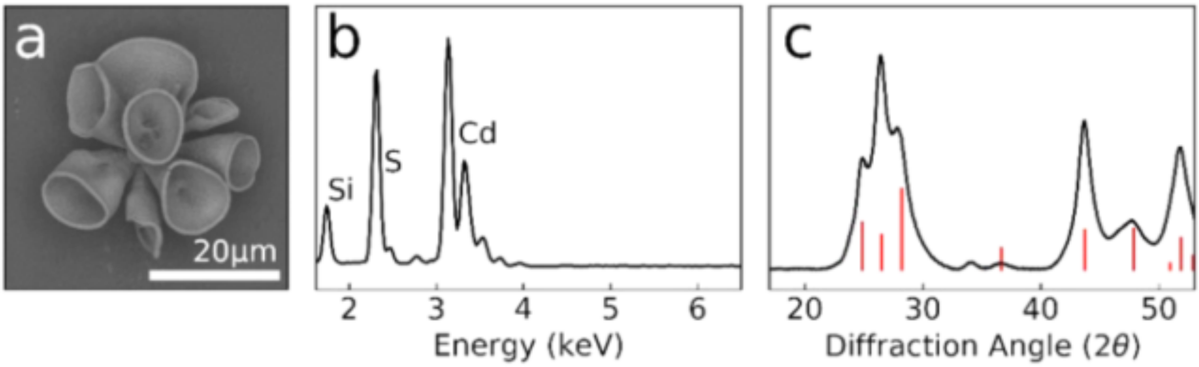


Figure S7: CdS nanocomposites. (A) SEM, (B) EDS, (C) XRD with reference lines depicted (COD 1011054).[1]

**S11 Removing the nanocrystals from the nanocomposites**

$CdCO_3$ nanocomposites were placed in a single zone tube furnace. The pressure was reduced to below 1 mbar and the furnace was heated to 500°C. After 24 hours the furnace was allowed to cool. The resulting structures were analysed with SEM and EDS and were found to consist only of silica (Figure S8).

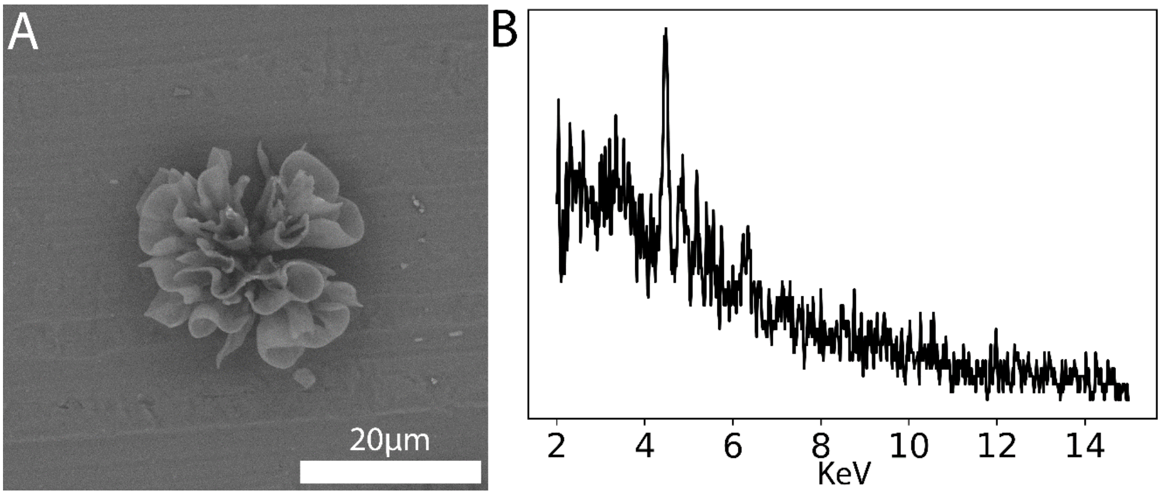


Figure S8: Empty structure. (A) SEM, (B) EDS showing absence of cadmium (3.1 KeV). The minor peak at 4.5 KeV corresponds to traces of barium from the initial composite.

**S12 Converting to $MnCO_3$ nanocomposites**

Anhydrous $MnCl_2$ (630 mg, 0.1 M) was dissolved in 50 mL degassed water. A substrate containing $BaCO_3$ nanocomposites was placed in this solution under nitrogen atmosphere for 60 minutes. Afterwards the substrate was removed from the solution and washed in two demineralized water baths followed by an acetone bath. The resulting nanocomposites were analysed with SEM, EDS, and XRD (Figure S9).

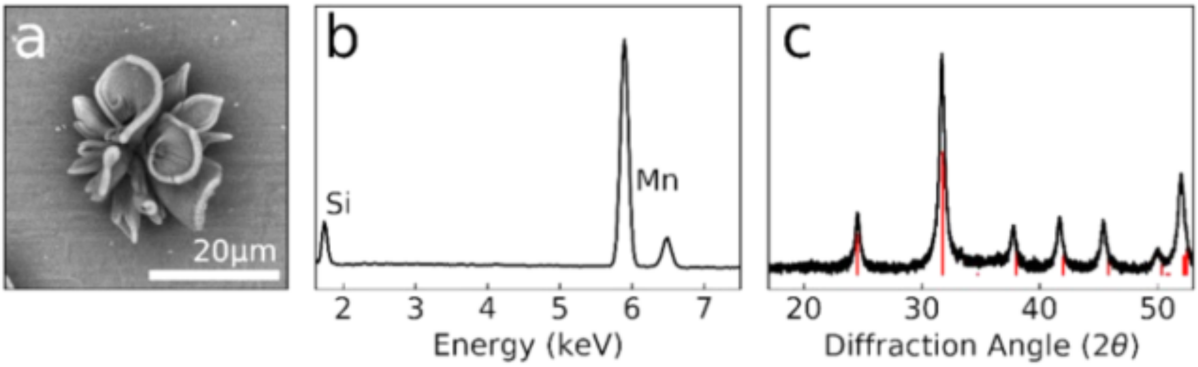


Figure S9: $MnCO_3$ nanocomposites. (A) SEM, (B) EDS, (C) XRD with reference lines depicted (COD 5000207). [1]

**S13 Converting to $Mn_3O_4$ nanocomposites**

$MnCO_3$ nanocomposites were placed in a single zone tube furnace. The furnace was purged of oxygen and filled with nitrogen and subsequently heated to 540°C for 13 hours. Afterwards, the furnace was allowed to cool. The resulting nanocomposites were characterized with SEM, EDS and XRD (Figure S10).

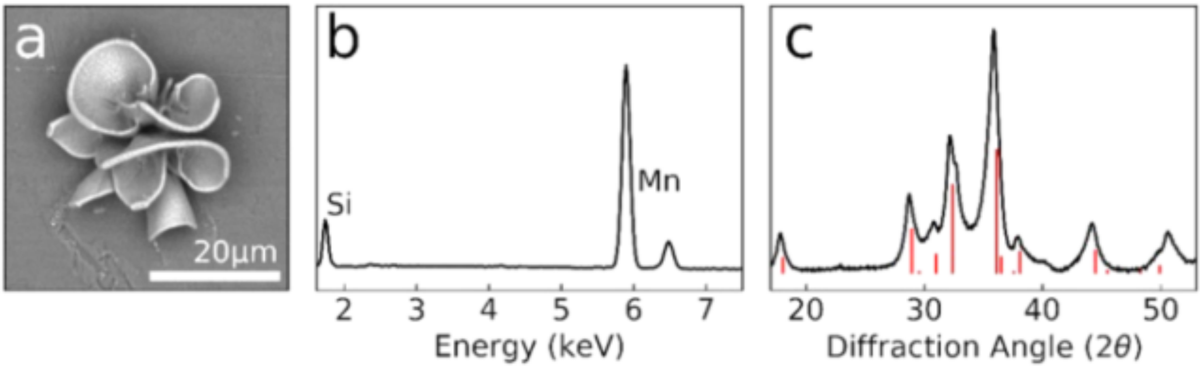


Figure S10: $Mn_3O_4$ nanocomposites. (A) SEM, (B) EDS, (C) XRD with reference lines depicted (COD 9001302).[1]

**S14 Converting to α-$Mn_2O_3$ nanocomposites**

$MnCO_3$ nanocomposites were placed in an open single zone tube furnace with the structures being exposed to air. The furnace was heated to 540°C for 24 hours. Afterwards, the furnace was allowed to cool. The resulting nanocomposites were characterized with SEM, EDS and XRD (Figure S11).

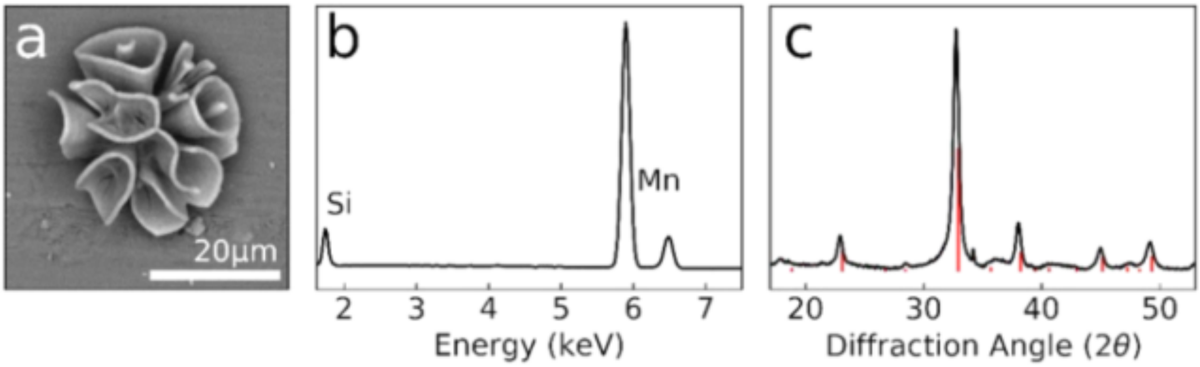


Figure S11: α-$Mn_2O_3$ nanocomposites. (A) SEM, (B) EDS, (C) XRD with reference lines depicted (COD 9007520).[1]

**S15 Converting to MnO nanocomposites**

$MnCO_3$ nanocomposites were placed in a single zone tube furnace. The furnace was purged of oxygen and filled with nitrogen with 5 vol% $H_2$. A flow of $H_2$ (5 mL $min^{-1}$) and $N_2$ (95 mL $min^{-1}$ for a total of 100 mL $min^{-1}$). After 4 hours the hydrogen flow was stopped and the oven was allowed to cool. The resulting nanocomposites were characterized with SEM, EDS and XRD (Figure S12).

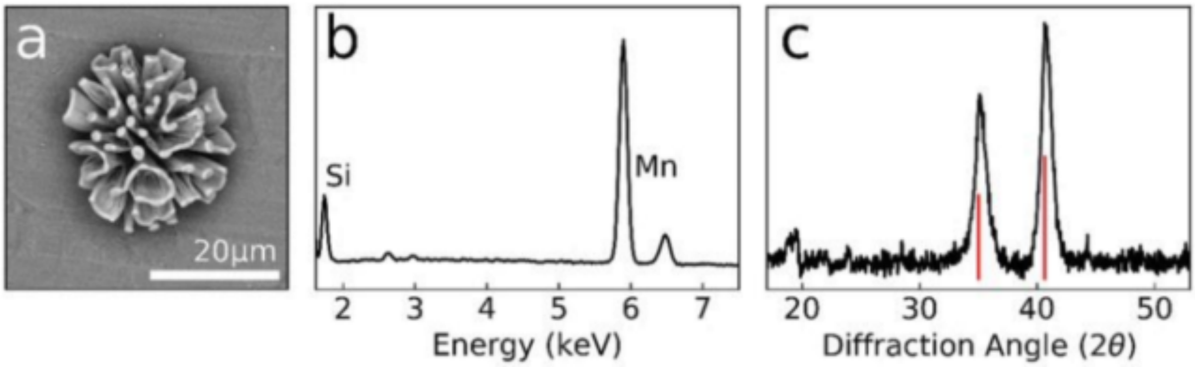


Figure S12: MnO nanocomposites. (A) SEM, (B) EDS, (C) XRD with reference lines depicted (AMCSD 0007528).[1]

**S16 Removing the nanocrystals from hydrogen treated nanocomposites**

$CdCO_3$ nanocomposites were placed in a single zone tube furnace. The pressure was reduced to below 5 mbar with a 15 sccm $H_2$ flow. The furnace was heated to 500°C for 4 hours after which the furnace was allowed to cool. The structures were transferred to another single zone tube furnace and re-heated to 500°C at pressures below 1 mbar for 24 hours. Afterwards the furnace was allowed to cool. The resulting structures were analysed with SEM and EDS and were found to consist only of silica (Figure S13). A small trace of iron from the substrate was detected in the EDS.

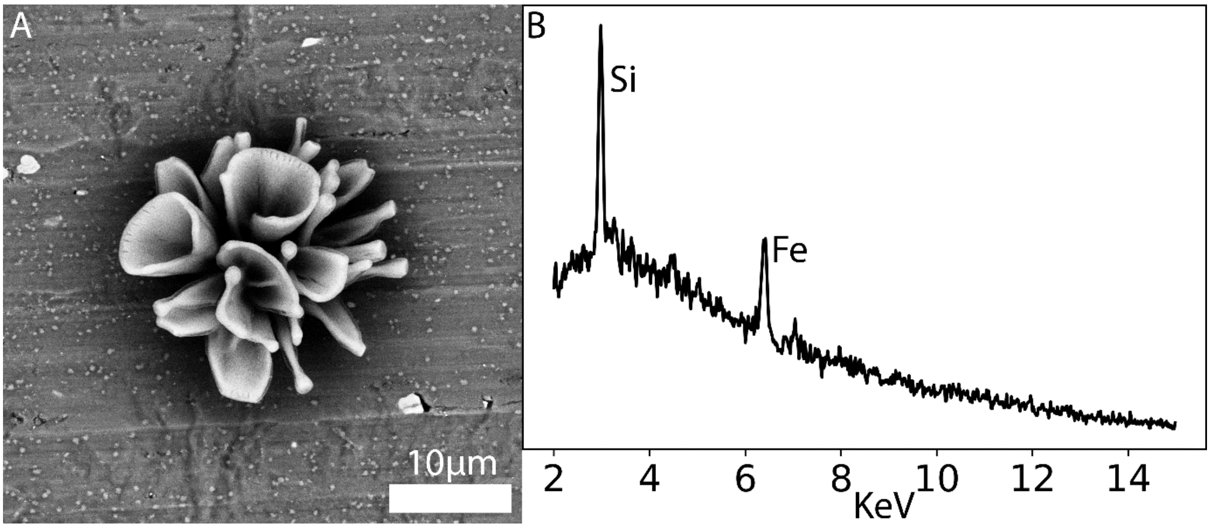


Figure S13: Hydrogen treated empty nanocomposites. (A) SEM, (B) EDS.

**S17 Lambda calculation**

We calculate λ using the following formula:

$$\lambda = \frac{Vol_{SiO_2}}{Vol_{SiO_2} + Vol_{BaCO_3}}$$

with $Vol_{SiO2}$ and $Vol_{BaCO3}$ the volumes of silica and barium carbonate in the original nanocomposite. Here we computed $Vol_{SiO_2}$ and $Vol_{BaCO_3}$ as follows:

$$V_{SiO_2} = at.\%_{SiO_2} * \frac{M_{r_{SiO_2}}}{\rho_{SiO_2}} = 0.2 * \frac{60.0843}{1.28 \pm 0.23}$$

$$V_{BaCO_3} = at.\%_{BaCO_3} * \frac{M_{r_{BaCO_3}}}{\rho_{BaCO_3}} = 0.8 * \frac{197.34}{4.29}$$

The atomic ratio is determined using EDS analysis to be 0.8:0.2 for barium carbonate versus silica. The density of the silica phase ($\rho_{SiO_2}$) from similar sol-gel reactions found in literature is 1.28±0.23 g/cm$^3$.[1,2] Substituting $Vol_{SiO_2}$ and $Vol_{BaCO_3}$ we calculate λ as follows

$$\lambda = \frac{0.2 * 60/(1.28 \pm 0.23)}{0.2 * 60/(1.28 \pm 0.23) + 0.8 * 197.34/4.29} = 0.20 \pm 0.03$$

**S18 Volume characterization**

All of the analyzed nanocomposites are coral-shaped with multiple extrusions. To calculate the azimuthal and radial length ratios of these composites, we developed an analysis program written in Python using the OpenCV Python library. As shown in Figure S14, we first find determine the contour Fig. R1A, red line) and determine the center point (Fig. R1B blue dot) of the initial structure by calculating the closest fit ellipse. Then, using the contour of the structure, we calculate the distance of each pixel to the center point using the Numpy Python library. Based on changes in the first and second derivative of this distance matrix we generate an initial selection of extremity pairs on the outside of the structure in the image plane (See Fig. S14B red dots). This initial selection is refined by hand, after which the program calculates the distance between each extremity pair (Fig. S14C green lines) as well as the distance of each selected point to the center point (Fig. S14D light blue lines going

towards center point). This process is repeated for the final structure that is left after all reactions have been performed. The closest matching extremity pairs before and after conversion are found and the final length is divided by the initial length to get the ratio of change. The average of all these ratios over multiple structures yields the radial and Azimuthal length ratios. The data points collected via this methodology are plotted in Figure S15 and the raw data is shown in Table S1.

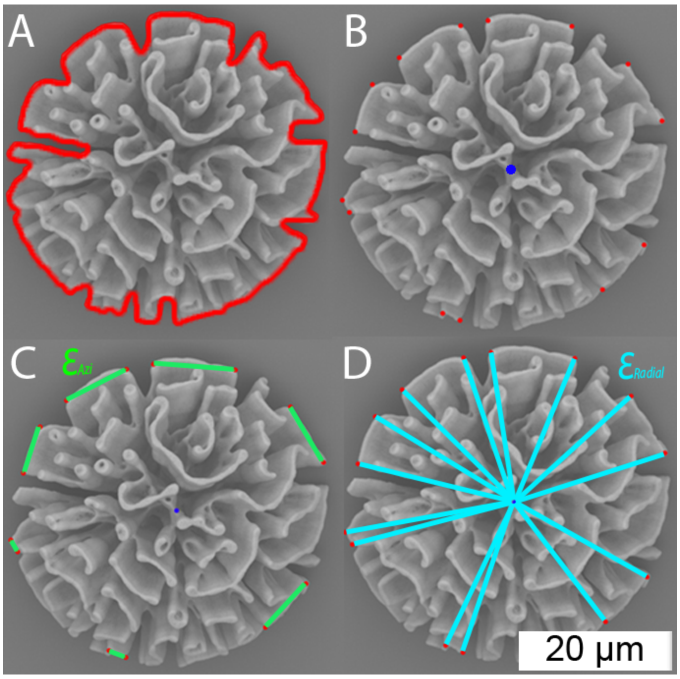


Figure S14: Geometrical analysis of a nanocomposite, here illustrated for a $BaCO_3/SiO_2$ shape. (A) The contour of the structure is determined and shown in red. (B) Using this contour, the far left and right points of extremities are determined and indicated as red dots. The center point is also calculated and shown in blue. (C) The azimuthal length ($\varepsilon_{Azi}$) is determined from the distance between the left and right points of extremities as shown in green. (D) The radial length ($\varepsilon_{Radial}$) is calculated from the distance of each extremity to the center point as shown in light blue.

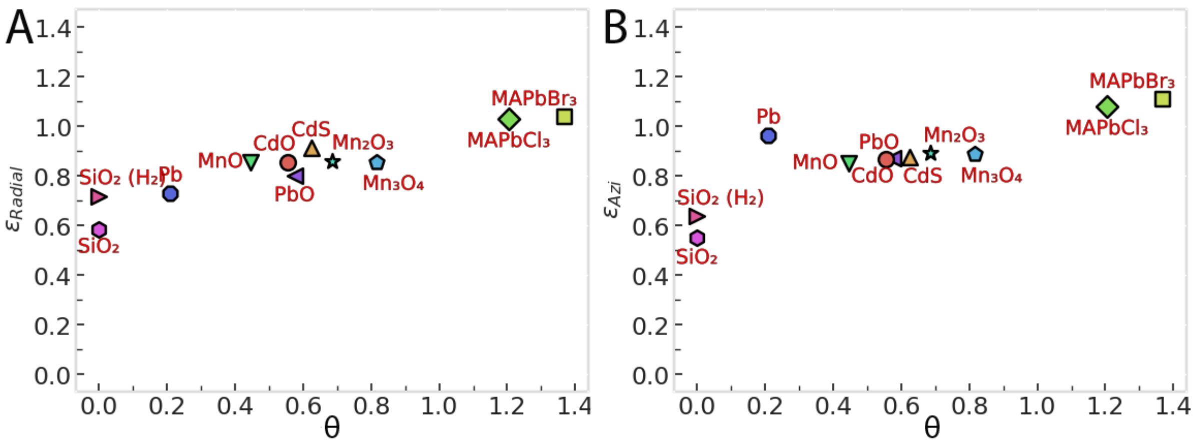


Figure S15: (A) plot of $\varepsilon_{Radial}$ against θ. (B) plot of $\varepsilon_{Azi}$ against θ.

| Material | $\varepsilon_{radial}$ | Stdev | $\varepsilon_{Azi}$ | Stdev | datapoints |
|---|---|---|---|---|---|
| $SiO_2$ (empty) | 58.9562 | 6.023602 | 54.78607 | 10.22078 | 6 |
| $SiO_2$ (empty) ($H_2$ treated) | 71.80882 | 5.66519 | 63.64384 | 11.34844 | 8 |
| MnO | 85.55695 | 6.747403 | 84.98738 | 7.178188 | 4 |
| $Mn_2O_3$ | 85.96444 | 2.248684 | 88.9602 | 5.31701 | 6 |
| $Mn_3O_4$ | 85.52985 | 4.949954 | 88.66581 | 5.379818 | 9 |
| $MAPbCl_3$ | 103.0633 | 1.821404 | 107.8634 | 7.204524 | 18 |
| $MAPbBr_3$ | 103.6395 | 2.036621 | 110.6781 | 8.148796 | 22 |
| PbO | 79.97242 | 1.588453 | 87.08794 | 10.87247 | 9 |
| Pb | 72.97473 | 2.141826 | 96.31311 | 15.92574 | 10 |
| CdO (multiple temperatures) | 86.5927 | 2.774178 | 81.18893 | 6.940238 | 45 |
| CdS | 91.18573 | 2.952639 | 87.44869 | 5.351502 | 12 |

| Material | Hydrogen | ε | ϕ | θ |
|---|---|---|---|---|
| $SiO_2$ (empty) | 0 | 17.46994381 | -3.16257024 | 0 |
| $SiO_2$ (empty) ($H_2$ treated) | 1 | 29.08643597 | 11.35804496 | 0 |
| MnO | 1 | 61.79654738 | 52.24568423 | 44.7 |
| $Mn_2O_3$ | 0 | 68.03155073 | 60.03943841 | 68.6 |
| $Mn_3O_4$ | 0 | 67.24036896 | 59.0504612 | 81.5 |
| $MAPbCl_3$ | 0 | 119.6572925 | 124.5716156 | 120.4 |
| $MAPbBr_3$ | 0 | 126.9547386 | 133.6934232 | 136.9 |
| PbO | 0 | 60.65356047 | 50.81695058 | 57.5 |
| Pb | 1 | 67.69292946 | 59.61616182 | 21 |
| CdO (multiple temperatures) | 0 | 47.63831059 | 34.54788824 | 55.6 |
| CdS | 0 | 69.73221824 | 62.1652728 | 62.4 |

Table S1: Raw data of volume change data in percentage points.

**S19 Spherical symmetry**

Figure S16 shows the side view of a nanocomposite after conversion. The distance from its nucleation point to the extremities was determined and it was found that this stayed mostly constant with a maximum variation of less than 7%. Indicating that the spherical symmetry is preserved after conversion.

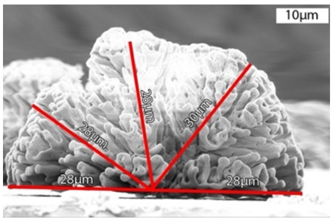


Figure S16: SEM images showing spherical symmetry after conversion. Showing side view with measured distances from the nucleation point.

**References**

(1) Hendrikse, H. C.; van der Weijden, A.; Ronda-Lloret, M.; Yang, T.; Bliem, R.; Shiju, N. R.; van Hecke, M.; Li, L.; Noorduin, W. L. Shape-Preserving Chemical Conversion of Architected Nanocomposites. *Adv. Mater.* **2020**, *32* (52), 1–7.

(2) Kellermeier, M.; Cölfen, H.; García-Ruiz, J. M. Silica Biomorphs: Complex Biomimetic Hybrid Materials from “Sand and Chalk.” *Eur. J. Inorg. Chem.* **2012**, No. 32, 5123–5144.
